# Surgical outcomes of primary intraocular lens implantation for the treatment of aphakia in pediatric cataracts in the Brazilian public health system

**DOI:** 10.5935/0004-2749.20230045

**Published:** 2023

**Authors:** Antonio Carlos Lottelli, Virgilio Figueiredo Silva, Marjorie Fornazier Nascimento Queiroz

**Affiliations:** 1 Department of Surgical Specialties and Anesthesiology, Ophthalmology Division, Faculdade de Medicina de Botucatu, Universidade Estadual Paulista “Júlio de Mesquita Filho”, Botucatu, SP, Brazil.; 2 Faculdade de Medicina de Botucatu, Universidade Estadual Paulista “Júlio de Mesquita Filho”, Botucatu, SP, Brazil.

**Keywords:** Cataract extraction, Intraocular lens, Intraoperative complications, Glaucoma, Anterior eye segment, Child, Extração de catarata, Lentes intraoculares, Complicações intraoperatórias, Glaucoma, Segmento anterior do olho, Criança

## Abstract

**Purpose:**

To evaluate primary intraocular lens implantation in the treatment of children’s aphakia in the Brazilian public health system and compare the outcomes among different age groups.

**Methods:**

Children aged 0-12 years old with unilateral or bilateral congenital/developmental cataracts and underwent primary intraocular lens implantation were included.

**Results:**

A total of 108 eyes from 68 children were evaluated, and the children were divided into four age groups (<7 months [mo]; 7 mo-2 years old [y/o]; 2-5 y/o, and >5 y/o) were evaluated. Nineteen eyes (17.59%) presented visual axis opacification as a postoperative complication, which was more frequently observed in the <7 mo age group (37.93%). The difference was significant between the <7 mo and >5 y/o age groups (p=0.002). Visual axis opacification was divided into two categories: pupillary membrane and lens cell proliferation. Eight eyes presented pupillary membrane, whereas 14 showed lens cell proliferation. Out of eight eyes with pupillary membrane, seven occurred in the <7 mo age group. The difference between the <7 mo age group and the 2-5 y/o or >5 y/o age group was significant (p=0.01). Lens cell proliferation was more frequent in the <7 mo and 2-5 y/o age groups, but the difference was significant only between the < 7 mo age group and >5 y/o age group (p=0.040). Glaucoma and glaucoma suspect cases were not observed during the follow-up period.

**Conclusions:**

The main complication found in the study was visual axis opacification, which had a higher incidence in children operated on or before the age of 7 months.

## Abstract (pt)

**Objetivo:**

Avaliar o implante de lente intraocular primária para tratamento da afacia pediátrica no Sistema Único de Saúde (SUS) e comparar os resultados em diferentes faixas etárias.

**Métodos:**

Foram incluídas crianças com catarata congênita e do desenvolvimento unilateral ou bilateral de 0-12 anos de idade e submetidas a implante de lente intraocular primária.

**Resultados:**

Cento e oito olhos de 68 crianças divididas em quatro grupos de idade (<7m; 7m-2a; 2-5a e > 5a) foram avaliados. Dezenove olhos (17,59%) apresentaram opacificação do eixo visual como complicação pós-operatória. Essa complicação foi mais frequente na faixa etária <7 meses (37,93%). A diferença foi significativa entre os grupos de idade <7 meses e > 5 anos (p=0,002). A opacificação do eixo visual foi dividida em duas categorias: membrana pupilar e proliferação de células do cristalino. Oito olhos apresentaram membrana pupilar e 14 proliferação de células do cristalino. Dos oito olhos com membrana pupilar, sete ocorreram na faixa etária <7 meses. A diferença entre o grupo de idade <7 meses e os grupos de 2-5 anos e > 5 anos foi significativa (p=0,01). A proliferação de células do cristalino foi mais frequente nos grupos de idade <7 meses e 2-5 anos, mas significativa apenas quando comparados o grupo de idade <7 meses com o grupo> 5 anos de idade (p=0,040). Glaucoma e suspeitos de glaucoma não foram observados durante o acompanhamento.

**Conclusões:**

A principal complicação encontrada no estudo foi a opacificação do eixo visual. Sua incidência foi maior em crianças operadas antes dos 7 meses de idade.

## INTRODUCTION

Cataract surgery in children has improved dramatically in recent decades. Modern surgical techniques and advances in the use of equipment and supplies and intraocular lenses (IOL) has allowed for implantation even in younger children^(1,2)^.

Despite the advances, some studies do not recommend implantation in children under 7 months old with unilateral cataracts due to the rate of visual axis opacification that requires new surgical interventions. Instead, the treatment of aphakia with contact lenses is recommended at this age group^(3)^. Implantation for bilateral cataracts in patients under 2 years old is also controversial, but no consensus on the standard procedure has been established^(4,5)^.

However, in developing countries, the treatment of aphakia with contact lenses is not a viable alternative for most of the population. Furthermore, the contact lenses suitable for aphakia treatment are not even available in the markets in most of these countries.

In addition, most studies have only evaluated age groups where the primary implant is controversial. However, where the implant is already considered a standard procedure, a comparison among age groups can better demonstrate the role of age in the outcomes.

This study sought to evaluate the intraoperative and postoperative outcomes of primary intraocular lens implantation for the treatment of children’s aphakia in the Brazilian public health system and to compare the outcomes among different age groups.

## METHODS

This is an interventionist retrospective study of pseudophakic eyes in patients who underwent surgery according to the protocol “Primary intraocular lens implantation for treatment of congenital and developmental cataract” (Appendix 1). The study was approved by the local Ethics Committee.

Patient enrollment was consecutive, and all children with unilateral or bilateral congenital/developmental cataracts aged 0-12 years old were considered. All eyes with a horizontal corneal diameter smaller than 10 mm, persistent fetal vasculature (PFV), or other ocular abnormalities were excluded.

Children diagnosed with congenital cataracts in the first weeks of life underwent surgery as soon as possible after the fourth week of life. In children with bilateral cataracts, the second eye was operated on between 1 and 2 weeks after the first surgery or on the same day surgery.

All patients were operated on by the same surgeon (ACL) who used the posterior approach for capsulotomy and vitrectomy. The same technique was used in all age groups (Appendix 1).

The intra- and postoperative outcomes were analyzed according to age group at surgery, type of complication, and time between surgery and the detection of postoperative complications.

The classification criteria for glaucoma and glaucoma suspect followed the criteria established in The Infant Aphakia Treatment Study (IATS)^(7)^.

Pre- and postoperative complications were compared among age groups based on the chi-square distribution for comparison of proportions, at a significance level of 5% (SAS for Windows, v.9.4).

## RESULTS

This study evaluated 108 eyes from 68 children, 35 (51.47%) of whom were male. Forty-three children (83 eyes) had bilateral cataracts, and in three of them, only one eye was operated on according to the protocol and included in the study. A further 25 children had unilateral cataracts. The ages at surgery and follow-up are shown in table 1.

**Table 1:** Age at surgery and at follow-up and number of eyes operated in each age group

Age group	No. of eyes	Mean ± SD age/surgery (min-max)	Mean ± SD age/follow-up (min-max)
**<7 mo**	29	0.27 ± 0.13 (0.11-0.54) y/o	2.32 ± 1.78 (0.27-5.33) y
**7 mo-2 y/o**	15	1.10 ± 0.38 (0.61-1.67) y/o	2.81 ± 2.04 (0.59-5.76) y
**2-5 y/o**	32	3.13 ± 0.52 (2.39-4.46) y/o	1.88 ± 1.47 (0.27-5.42) y
**>5 y/o**	32	6.78 ± 1.84 (5.01-12.20) y/o	2.60 ± 1.65 (0.46-4.58) y
**Total**	108	3.16 ± 2.80 90.11-12.20) y/o	2.34 ± 1.70 (0.27-5.76) y

SD= standard deviation.

A single-piece aspheric acrylic hydrophobic IOL (Alcon AcrySof^®^ IQ) was implanted in the bag except in two eyes with intraoperative complications as subsequently described here.

Three eyes (2.77%) presented posterior capsule rupture as an intraoperative complication. One occurred in the <7 mo age group, in the right eye of a 3-month-old female baby with bilateral cataracts during a single-piece IOL injection. The IOL was explanted, and a three-piece IOL (Sensar^®^ AR40e) was placed in the ciliary sulcus with optic capture in the capsulorhexis. The other two occurred in the 2-5 y/o age group, both of them with polar posterior cataracts, one in the right eye of a 3.9-year-old male child with bilateral cataracts in which a posterior capsule rupture occurred during nucleus aspiration. A three-piece IOL was implanted in the ciliary sulcus with optic capture in the capsulorhexis. The other occurred in the left eye of a 2.7-year-old female child with a unilateral cataract during a single-piece IOL injection. The same IOL was maintained and captured reversely in the capsulorhexis (haptics in the bag and optic in the sulcus). Thus, 4% of eyes in children with unilateral cataracts and 2.4% of those with bilateral cataracts had posterior capsule rupture as an intraoperative complication. No significant differences were observed among the age groups (Table 2).

**Table 2:** Intraoperative (PCR) and postoperative (VAO) outcomes, and average IOP according to age group

Age group	No. eyes	PCR(%)	VAO (%)	Aver. IOP ± SD (min-max) (mmHg)
**<7 m**	29	1 (3.45)	11 (37.93)	12.55 ± 3.78 (6-19)
**7m-2 y/o**	15	0	2 (13.33)	14.84 ± 2.85 (9-18)
**2-5 y/o**	32	2 (6.25)	5 (15.63)	13.03 ± 2.35 (10-19)
**>5 y/o**	32	0	1 (3.12)	12.91 ± 2.26 (9-18)
**Total**	108	3 (2.77)	19 (17.59)	13,04 ± 2.87 (6-19)

PCR= Posterior capsule rupture; VAO= Visual axis opacification; Aver. IOP= average IOP.

Moderate vitreous bleeding from sclerotomy was also observed as an intraoperative complication in a 2.8-year-old female child with a unilateral cataract in the left eye, which resolved spontaneously within 15 postoperative days.

None of the eyes that presented intraoperative complications showed postoperative complications during the follow-up. No significant differences were noted in the intraoperative complications among the age groups.

During follow-up, 19 eyes (17.59%) presented visual axis opacification as a postoperative complication and required further surgery to clear the visual axis. This complication was more frequent in the <7 mo age group (37,93%). Furthermore, the difference was significant between the <7 mo age group and >5 y/o age group (p=0.002) but not between the <7 mo age group and the 7 mo-2 y/o (p=0.178) or 2-5 y/o (p=0.097) age groups. No significant differences were observed among the other groups (Table 2).

The average IOP observed at the last examination in the different age groups was 13.04 mmHg (Table 2). Glaucoma and glaucoma suspect cases, as categorized according to the adopted criteria, were not observed during the follow-up.

Visual axis opacification was divided into two categories: pupillary membrane and lens cell proliferation on the posterior surface of the IOL. Eight eyes presented pupillary membrane, whereas 14 showed lens cell proliferation. Three eyes presented both categories of complications, with one at the same time and the two others at different times (Table 3).

**Table 3:** Incidence of pupillary membrane and lens cell proliferation per age group

Age Group	No. eyes	PM (%)	Aver. time ± SD (min-max)	LCP (%)	Aver. time ± SD (min-max)
**<7 m**	29	7 (24.13)	2.38 ± 1.99 m (0.26-6.05 m)	7 (24.13)	3.48 ± 3.50 m (3.02-12.6 m)
**7m-2 y/o**	15	1 (6.67)	0.7 m	1 (6.67)	5.72 m
**2-5 y/o**	32	0	-	5 (15.63)	9.70 ± 2.87 m (6.67-13.56 m)
**>5 y/o**	32	0	-	1 (3.13)	15.12 m
**Total**	108	8 (7.40)	1.81 ± 1.94 m (0.26-6.05 m)	14 (12.96)	6.08 ± 4.45 m (3.02-15.12 m)

PM= pupillary membrane; Aver. time= average time after surgery; LCP= lens cell proliferation.

Out of eight eyes with pupillary membrane, three were from unilateral cataract cases (12%), and five were from bilateral cataract ones (6%). Furthermore, it was observed in 24.13% of eyes in the <7 mo age group. The difference between the <7 mo and 2-5 y/o or >5 y/o age groups was significant (p=0.01). On average, this complication was detected and resolved 1.3 months postoperatively. Out of 14 eyes with lens cell proliferation, two were in unilateral cataract cases (8%), whereas 12 were from bilateral cataract cases (14.45%). This complication was more frequent in the <7 mo and 2-5 y/o age groups but significant between the <7 mo age group and the >5 y/o age group (p=0.040). Lens cell proliferation was detected and resolved on an average of 6.8 months postoperatively (Table 3).

Other complications, such as vitreous tag, IOL displacement or important decentration, and other severe complications, such as endophthalmitis and retinal detachment, were not detected during the follow-up. Visual acuity, fusional ability, and other motility disorders were not assessed in this study.

## DISCUSSION

In the present study, intraoperative complications occurred in 2.77% of the eyes, and visual axis opacification occurred as postoperative complications in 17%. The incidence of visual axis opacification in the eyes of patients <7 mo old was 37.04% and significantly higher compared with the >5 y/o age group. On average, pupillary membrane was detected 1.8 months after surgery, whereas lens cell proliferation was detected 6.08 months after surgery. No eyes developed glaucoma during the average 2.42 years of follow-up.

The sample was divided into four groups, two with ages where the primary implant is controversial in comparison with the literature^(3-4)^, mainly in the <7 mo age group, and two where the implant has consensus. In older pediatric patients, vitrectomy is not necessary and capsulotomy can be performed with yag-laser (>5 y/o age group). Despite this, the same technique was used in all patients^(8)^.

Several techniques are described for cataract surgery in children considering the basic steps of posterior capsule opening and anterior vitrectomy. The chosen technique, with capsulotomy and vitrectomy performed by *pars plana/plicata*, is easier than the other techniques, mainly in babies in their first few weeks of life. At this age, if posterior capsulorhexis is performed before the IOL, implanting this “relatively large” IOL between the remnants of the anterior and posterior capsule bags is difficult, with a risk of IOL dislocation into the vitreous. If the choice is to implant the IOL and perform the capsulotomy and anterior vitrectomy after, the tightened IOL inside the small bag makes its displacement to gain access to the posterior capsule and vitreous a tough task. The techniques that use the anterior approach for vitrectomy could also be associated with vitreous attachment in the anterior segment and pupillary irregularity after surgery if vitreous removal is not complete. An incidence of 28% of corectopia was described in patients who underwent surgery before 7 months of age using different techniques^(3)^. Comparing the anterior and posterior approaches for capsulotomy and vitrectomy in pediatric cataracts with primary IOL implant, in patients aged 3 months to 9 years old, the authors found more complications in the anterior approach^(9)^. No corectopia or vitreous tag was detected as a postoperative complication in this study.

The disadvantage of using the pars *plana/plicata* for capsulotomy and vitrectomy is the need to use an additional opening. Devices such as small 25- or 27-gauge vitrector probes could also allow this procedure using transconjunctival sutureless vitrectomy^(10)^, but they were not used in the present study. Vitreous bleeding was observed in one patient at the time of surgery but was resolved within 15 postoperative days. No posterior segment complications were observed postoperatively during the follow-up period.

Three patients had posterior capsule rupture as an intraoperative complication, one in the <7 mo age group and two in the 2-5 y/o age group both with polar posterior cataracts. In very young patients, IOL insertion is a critical surgical moment because of the small size of the eye and bag, whereas polar posterior cataracts increases the risk for posterior capsule rupture in any age group^(11)^.

Regarding postoperative complications, visual axis opacification is the postoperative complication responsible for the largest number of reinterventions in pediatric cataract surgeries with primary IOL implantation, reaching 68% in unilateral^(3)^ and 32% in bilateral cataracts^(12)^ in children aged 1 to 7 months of age at surgery and within a 5-year follow-up. However, this complication frequently occurs in the first year of follow-up^(13)^. In the present study, visual axis opacification occurred in 37.04% of the <7 mo age group. Despite the average follow-up of 2.42 years, the present study included patients who did not complete the first year of follow-up. Considering only 18 cases operated on in the <7 mo age group that completed at least a 1-year follow-up, the visual axis opacification incidence was 44.44%.

The incidence of visual axis opacification in the <7 mo age group (37.93%) was around two-fold greater than those in the 7 mo-2 y/o (13.33%) and 2-5 y/o (15.63) age groups. However, the difference was only significant when compared with the >5 y/o age groups (3.12%). The incidence of visual axis opacification in the 7 mo-2 y/o and 2-5 y/o age groups was quite similar.

Visual axis opacification can be divided into two distinct categories by origin: pupillary membrane formation or proliferation of epithelial lens cells. Pupillary membrane occurs due to the great inflammatory activity presented in pediatric eyes^(14)^. Regarding lens cell proliferation, despite the removal of the posterior capsule and the anterior vitreous, lens epithelial cells are able to grow on the posterior surface of the IOL. Lens cell proliferation can also occur in aphakic eyes with growth of lens epithelial cells towards the visual axis; however, in aphakia, it is less frequent because there is fusion of the remaining lamellae of the anterior and posterior capsule, trapping the lens epithelial cells.

The IATS study presented pupillary membrane in 28% and lens cell proliferation in 40% of the eyes with primary IOL implant with five-year follow-up. In the present study seven of the eight eyes that developed pupillary membrane belonged to the < 7 mo age group (24.13%) and accounted for half of the cases of visual axis opacification in this group. The only other case with pupillary membrane occurred in a 7.38 mo boy with unilateral cataract included in the 7 mo-2 y/o age group. These findings suggest that the higher incidence of visual axis opacification in the < 7 mo age group is partly due to the high inflammatory activity present in very young children.

In the IoLunder2 study, the incidence of pupillary membrane and lens cell proliferation were 13% and 32%, respectively, in children operated on in the first 2 years of life and with five-year follow-up. Considering only children at this age in the present study, the incidence of pupillary membrane is 18% and of lens cell proliferation is also 18%. A recent report of the IoLunder2 study showed a relationship between low socioeconomic level and higher incidence of pupillary membrane^(13)^. The incidence of this complication in our study is lower than that in the IATS study (considering only <7 m age group), but higher than that in the IoLunder2 study (considering <7 m and 7m-2 y/o age groups), even though the low socioeconomic level is the condition of the majority of the patients in our sample. Related to lens cell proliferation, the low incidence compared with the IATS and IoLunder2 studies could in part be related to the shorter follow-up as mentioned above. Careful aspiration of the lens epithelial cells attached to the edge of the anterior capsule is an attempt to reduce this complication, but this aspiration can be difficult if there is no good pupillary dilation, which is common in young babies. It could also be one of the factors that contribute to the greater incidence of this complication in this age group, but there are no studies about it. Pupillary membrane was detected and resolved on average 1.81 ± 1.94 m (0.26 - 6.05 m) and Lens cell proliferation 6.08 ± 4.45 m (3.02 - 15.12 m) after surgery.

Glaucoma is a common complication reported in children undergoing cataract surgery, mainly operated on before one year of age and particularly with few weeks of life^(7)^. The IATS study reports 7% and 14% glaucoma or glaucoma suspect in the first and fifth follow-up year respectively, excluding patients with corneal diameter less than 10 mm in which the incidence of glaucoma is significantly higher, but including patients with persistent fetal vasculature (PFV). PFV presented a risk 3.1 higher than eyes without this condition in the first follow-up year^(7-15)^. In the IoLunder2 study, the incidence of glaucoma or persistent ocular hypertension was approximately 30%; however, in this study, one-third of the patients had some ocular anomaly, including corneal diameter less than 10 mm and PFV^(16)^. In the present study, no children had glaucoma or glaucoma suspect during follow-up. This absence may be associated with the exclusion of patients with corneal diameter less than 10 mm and any associated abnormality like PVF, and still has an average follow-up of 2.4 years. A possible protective effect of IOL against glaucoma remains controversial. Systematic reviews show a protective effect^(17,18)^, but the most recent one found no significant difference between the primary IOL implant and aphakic groups^(19)^.

In studies such as IATS^(3,14,15)^ and IoLunder2^(4,13)^, the visual acuity achieved was similar treating aphakia with primary implant or contact lenses, but the number of reinterventions was higher in those with primary implant. However, the conditions of these studies are far from the reality of children in most countries mainly considering children assisted by the Public Health System. The children’s families do not have socioeconomic conditions to support the treatment with contact lenses and, in addition, the most commonly used lens for aphakia in USA and Europe (*SilSoft*^®^, Bausch and Lomb Rochester, NY), are not even available on their markets, including the Brazilian one. The *SilSoft*^®^ is a silicone contact lenses available up to +32 diopters that allows the children to keep the lens in the eye for up to a week, which is very comfortable for them and their parents. The lens available for this kind of use in Brazil goes up to +15 diopters. Regarding the visual acuity, a recent meta-analysis comparing primary IOL implantation and aphakia in patients with cataract younger than two years showed a significantly better visual acuity in the primary implant group^(19)^.

However, when the primary implant is performed, especially in children under one year old, the referral centers assume most responsibility in the children treatment. For this, these centers must have adequate facilities with experienced surgeons to perform the surgeries and solve complications as soon as they are detected. Pediatric cataracts are the biggest cause of preventable blindness in childhood^(20)^. In developing countries, specialized centers should be these children’s “lifeline”.

For patients operated on younger than seven months old, the number of surgical interventions, adopting the primary implant or aphakia, is similar in long-term follow-up^(21)^, because the aphakic children will have to undergo a new surgical intervention to implant the IOL. Thus, the expenses with extra interventions to solve the Visual Axis Opacification in the primary implant will not impact the treatment costs of the reference centers.

The limitations of this study are the relatively short follow-up and the lack of information regarding the patients’ visual development; however, these children will continue to be followed, and these data will be reported in future studies.

In conclusion, the main complication found in the study was visual axis opacification. Its incidence was higher in children operated on before the age of 7 months. The pupillary membrane was responsible for 50% of this complication in this age group and the lens cell proliferation for another 50%.

## Appendix 1 Primary Intraocular Lens Implant for Treatment of Congenital and Developmental Cataract Protocol

**Table:**

The local institutional Ethics Committee approved this protocol that sequentially included children (aged 0 to 12 years old) with congenital or developmental unilateral and bilateral cataract.
Eyes with horizontal corneal diameters smaller than 10 mm, persistent fetal vasculature, or other ocular anomalies were excluded.
Children with congenital cataracts diagnosed during the first weeks of life were operated on between the 5th and 6th weeks of life, and in bilateral cataracts, the second eye was operated within 1 to 2 weeks after the first eye, if not on the same day.
After anesthesia, keratometry (K) (Retinomax K-plus 2^®^, Righton, Tokyo, Japan), tonometry (Tono-Pen XL^®^, Reichert^®^ Technologies, Buffalo, USA), immersion ultrasound biometry, and pachymetry (OcuScan RxP^®^, Alcon, Fortworth, USA) were performed. Deuring the surgery, if possible, a single-piece hydrophobic acrylic intraocular lens (IOL) was implanted (Alcon *AcrySof^®^ IQ, Alcon, Fortworth*) in the bag, and the power as adjusted to minimize myopia in adulthood^(6)^. The posterior capsule is opened, and an anterior vitrectomy is performed via *pars plana/plicata* after IOL implantation.
In children younger than 1 year, an examination under anesthesia was scheduled every 3 months during the first year of life. In children older than 1 year, the examination was scheduled every 6 months.
In the examination, automated refraction, keratometry (K), tonometry, immersion ultrasound biometry, and pachymetry were performed, using the same instruments mentioned above.
Collaborative children older than 4 years of age were examined in the office. For these children, K and automated-refraction measurements were taken using an automated tabletop refractometer and keratometer (Potec PRK-6000^®^, Potec, Daejon, Korea), and AL measurements were performed using an optical biometer (IOL Master 500^®^, Zeiss, Jena, Germany). IOP was measured using a Goldman tonometer coupled with a slit-lamp.
**Surgical technique:**
Incisions of 1.50 mm wide and 1.5 mm long were performed at 10 and 2 o’clock perilimbals in the clear cornea. Trypan blue was injected into anterior chamber, followed by ophthalmic viscoelastic device (OVD). After that, a capsulorhexis of approximately 5.5 mm was performed using coaxial microforceps. Lens material was aspirated using separate irrigation/aspiration, and the lens epithelial cells from the capsulorhexis rim were removed. OVD was injected in the capsular bag and anterior chamber. The 10 o’clock incision was then enlarged to 2.4 mm. The IOL was injected in the capsular bag. One stitch was performed in a 10 o’clock incision with 10-0 absorbable suture (poliglactin 910, Vicryl^®^). Then, the conjunctiva was opened at 2.5 mm (children under 2 years old) or 3 mm (children older than 2 years old) from the limbus at 10 o’clock, followed by 1 mm sclerotomy. The anterior chamber OVD was aspirated by separate irrigation/aspiration. Anterior irrigation was maintained through the 2 o’clock anterior incision, and anterior vitrectomy and posterior capsule opening were performed using a 23-gauge vitrector through the sclerotomy. Finally, all incisions were closed using 10-0 absorbable sutures.
The children received intravenous hydrocortisone (Flebocortid^®^ 100mg) 10mg/kg during the surgery and dexamethasone 2mg/ml, 0.2ml subconjunctival immediately after surgery. Gatifloxacin 0.3% (Zymar^®^ Allergan) and prednisolone 1% (pred fort Allergan^®^) eye drops were applied every 4 h for 15 days, and after that, prednisolone 1% every 6h for a further 15 days. Oral prednisolone (1mg/ml) was also used for 5 days after surgery.
